# Predictors of intubation and mortality in COVID-19 patients: a retrospective study

**DOI:** 10.1186/s44158-021-00016-5

**Published:** 2021-11-27

**Authors:** Tiziana Cena, Gianmaria Cammarota, Danila Azzolina, Michela Barini, Simona Bazzano, Domenico Zagaria, Davide Negroni, Luigi Castello, Alessandro Carriero, Francesco Della Corte, Rosanna Vaschetto

**Affiliations:** 1 Anestesia e Terapia Intensiva, Azienda Ospedaliero Universitaria “Maggiore della Carità”, Novara, Italy; 2 Dipartimento di Medicina Traslazionale, Università del Piemonte Orientale, Novara, Italy; 3 Radiologia, Azienda Ospedaliero Universitaria “Maggiore della Carità”, Novara, Italy; 4 Medicina d’Urgenza, Azienda Ospedaliero Universitaria “Maggiore della Carità”, Novara, Italy

**Keywords:** COVID-19, Intubation, Factor risk, Mortality, Quantitative computerized tomography, Random forest

## Abstract

**Background:**

Estimating the risk of intubation and mortality among COVID-19 patients can help clinicians triage these patients and allocate resources more efficiently. Thus, here we sought to identify the risk factors associated with intubation and intra-hospital mortality in a cohort of COVID-19 patients hospitalized due to hypoxemic acute respiratory failure (ARF).

**Results:**

We included retrospectively a total of 187 patients admitted to the subintensive and intensive care units of the University Hospital “Maggiore della Carità” of Novara between March 1st and April 30th, 2020. Based on these patients’ demographic characteristics, early clinical and laboratory variables, and quantitative chest computerized tomography (CT) findings, we developed two random forest (RF) models able to predict intubation and intra-hospital mortality. Variables independently associated with intubation were C-reactive protein (*p* < 0.001), lactate dehydrogenase level (*p* = 0.018) and white blood cell count (*p* = 0.026), while variables independently associated with mortality were age (*p* < 0.001), other cardiovascular diseases (*p* = 0.029), C-reactive protein (*p* = 0.002), lactate dehydrogenase level (*p* = 0.018), and invasive mechanical ventilation (*p* = 0.001). On quantitative chest CT analysis, ground glass opacity, consolidation, and fibrosis resulted significantly associated with patient intubation and mortality. The major predictors for both models were the ratio between partial pressure of arterial oxygen and fraction of inspired oxygen, age, lactate dehydrogenase, C-reactive protein, glycemia, CT quantitative parameters, lymphocyte count, and symptom onset.

**Conclusions:**

Altogether, our findings confirm previously reported demographic, clinical, hemato-chemical, and radiologic predictors of adverse outcome among COVID-19-associated hypoxemic ARF patients. The two newly developed RF models herein described show an overall good level of accuracy in predicting intra-hospital mortality and intubation in our study population. Thus, their future development and implementation may help not only identify patients at higher risk of deterioration more effectively but also rebalance the disproportion between resources and demand.

**Supplementary Information:**

The online version contains supplementary material available at 10.1186/s44158-021-00016-5.

## Introduction

Severe acute respiratory syndrome coronavirus 2 (SARS-CoV-2) causes a wide spectrum of clinical manifestations, named coronavirus disease 19 (COVID-19), which range from asymptomatic infections to severe interstitial pneumonia. Unfortunately, the COVID-19 pandemic has led to a large number of critically ill patients in a very short time and is currently overwhelming the healthcare systems worldwide [1].

In this scenario, delays in intensive care unit (ICU) transfers [2–4] and in patient intubations [5, 6] have been associated with increased mortality among COVID-19 patients with hypoxemic acute respiratory failure (ARF). Thus, correct triage and prompt ICU allocation of patients scheduled to receive intubation in case of oxygen therapy or noninvasive ventilation failure are crucial to achieve an effective COVID-19 pandemic response [7].

Potential prognostic factors of intra-hospital mortality and intubation in patients hospitalized due to COVID-19-related hypoxemic ARF include older age [8, 9], male gender [9], diabetes [9], prothrombin time and D-dimer level [10], lymphocytopenia [11–13], leukopenia [11–13], C-reactive protein [11–13], lactate dehydrogenase [11–13], sequential organ failure assessment (SOFA) score [14], arterial oxygen pressure on inspired oxygen fraction ratio (PaO_2_/FiO_2_) [8, 14], and acute kidney injury [14].

The Fleischner Society Statement on Chest Imaging and COVID-19—issued on April 7th, 2020—recommends chest computerized tomography (CT) imaging for the triage of (*i*) patients with suspected COVID-19 presenting with moderate to severe clinical features and a high pretest probability of disease, (*ii*) COVID-19 patients with worsening respiratory status, and (*iii*) patients with functional impairment and/or hypoxemia after recovery from COVID-19 [15, 16]. Fittingly, a single-center study has recently shown how the compromised lung volume estimated by quantitative CT analysis is a strong predictor of the need for oxygenation support and intubation among COVID-19 patients [17]. Thus, an algorithm combining demographic and early clinical characteristics together with laboratory findings and chest CT analysis results may favor the early identification of COVID-19 patients requiring invasive mechanical ventilation (IMV) or at increased risk of mortality.

Our primary aim was to retrospectively identify the risk factors associated with intubation and intra-hospital mortality in a cohort of COVID-19 patients admitted to the hospital for hypoxemic ARF by analyzing their demographic characteristics, early clinical and laboratory variables, and quantitative chest CT analysis results. As a secondary endpoint, we sought to determine the performance of a newly developed algorithm based on the aforementioned variables in predicting the probability of IMV and intra-hospital mortality in our study population.

## Methods

### Patients and data

The present investigation is an observational retrospective single-center study. Ethical approval was issued by the Comitato Etico Interaziendale Novara, Italy (Chairperson Prof. G. Zulian) on May 20th, 2020 (Ethics Committee No. CE 121/20). The requirement for informed consent was waived due to the retrospective nature of the study.

We analyzed 187 consecutive patients with COVID-19 pneumonia diagnosed with real-time reverse transcriptase-polymerase chain reaction (RT-PCR) nasopharyngeal swabs, subjected to chest CT images, and admitted to the subintensive and intensive care units of our hospital between March 1st and April 30th, 2020. Patients with poor-quality chest CT images were excluded. The study was reported in accordance with STROBE guidelines.

### Clinical and laboratory characteristics

Demographic information, body mass index (BMI), time from first-symptoms, comorbidity, date of admission to hospital, clinical laboratory on admission including the first PaO_2_/FiO_2_ ratio, arterial oxygen saturation (SpO_2_), blood cell counts (i.e., leukocyte and lymphocyte count), biomarkers of inflammation (i.e., lactate dehydrogenase, ferritin, C-reactive protein, procalcitonin, fibrinogen), glycemia, and troponin were collected. Furthermore, we recorded the type of oxygen assistance administered—i.e., standard oxygen therapy (low-flow oxygen nasal cannula, Venturi mask, non-rebreathing mask), noninvasive ventilation [continuous positive airway pressure (CPAP) or bilevel positive airway pressure (BiPAP)], or invasive mechanical ventilation. All data were derived from both electronic hospital records and digitization of paper documents.

### Criteria for intubation

Criteria for intubation were cardiac or respiratory arrest; inability to protect the airway; coma or psychomotor agitation; unmanageable secretions or uncontrolled vomiting; life-threatening arrhythmias or electrocardiographic signs of ischemia; hemodynamic instability, defined as systolic arterial pressure < 90 mmHg despite adequate filling or use of vasoactive agents; intolerance to all interfaces; dyspnea during noninvasive ventilation administered as CPAP or BiPAP; respiratory rate > 30 breaths/min; SpO_2_ < 92% during CPAP or BiPAP; and acidosis.

### Quantitative CT analysis

**Fig. 1:**
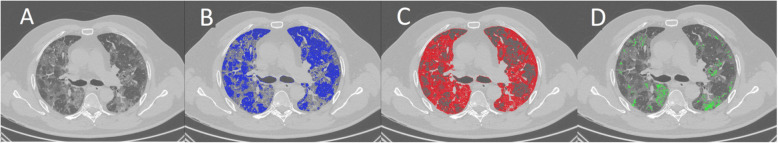
Quantitative CT analysis of a 74-year-old male COVID-19 patient. **a** Non-contrast chest CT on admission, showing a characteristic bilateral and subpleural ground glass opacity (GGO). **b** Well-aerated parenchyma segmented semi-automatically by a 3D slicer; the blue area is the result of the subtraction of the all parenchyma (HU − 1100; − 250) with GGO (− 700; − 250) + consolidation (− 250; + 150). **c** The GGO area, obtained by semi-automatic segmentation (HU − 700; − 250). **d** Manual segmentation of consolidation areas (HU − 250; + 150)

CT scan was performed within 1 day from admission. CT images were independently reviewed by two radiologists with 10 and 14 years of clinical experience: all radiologists were blinded to the clinical status of the patients. The lung parenchyma segmentation was performed through a software-based evaluation on a dedicated workstation using the open-source 3D Slicer software (Fig. 1). More details can be found in the Supplementary Information.

### Statistical analysis

A sample size was computed to ensure a predictive ability of the RF model close to 0.8 with a margin of error in the sample estimates *d* = 0.05. More details can be found in the Supplementary Information. Descriptive statistics were reported as median and interquartile range for continuous variables and percentages (absolute numbers) for categorical variables. Missing values were handled leaving null the estimate. The logistic regression model, odds ratio (OR) together with the 95% confidence intervals (95% CI), and *p* values were reported for each predictor, considering separately their association with intubation and intra-hospital mortality. The analyses were performed using R software (version 0.2) with the packages caret and rms.

### Random forest predictive tool

#### Model

To identify predictors of intra-hospital intubation or mortality, a random forest (RF) algorithm was employed. The variables having less than 10% of missing values were included in the predictive tool. More details can be found in the Supplementary Information.

## Results

### Patient characteristics

The main characteristics of the 187 patients included in the study are summarized in Table 1. One hundred forty patients (∼75%) were males, with a median age of 64 years, a median BMI of 28 kg/m^2^, and a median PaO_2_/FiO_2_ of 258 mmHg on admission. The median symptom onset was 7 days prior to admission. The most frequent comorbidities were hypertension (51%) and diabetes (24%). Main laboratory and chest CT findings on admission are also reported in Table 1. Forty-five patients (24%) received standard oxygen therapy, 86 patients (46%) received noninvasive ventilation, and 56 patients (30%) were admitted to ICU and required IMV.

**Table 1:** Patient characteristics and clinical, laboratory, and CT findings relative to alive and death patients

Variable	Valid cases	Alive *n*=129	Death *n*=58	Total	OR (univariable)	Cut point	AUC
Age (year)		62 (55–69)	71 (63–78)*	64 (57–72)	2.42 (1.54–3.97, *p*< 0.001)	65	0.69
Sex							
Male	140	90 (70%)	50 (86%)	140 (75%)	-	-	
Female	47	39 (30%)	8 (14%)*	47 (25%)	0.37 (0.15–0.82, *p*=0.019)	-	
PaO_2_/FIO_2_ ratio	181	267 (227–306)	217 (168–266)*	258 (208–309)	0.48 (0.30–0.75, *p*=0.002)	241	0.68
SpO_2_ (%)	187	92 (89–95)	87 (80–93)*	91 (87–95)	0.63 (0.47–0.84, *p*=0.002)	88.4	0.66
Symptom onset	177	7 (5–10)	7 (4–9)	7 (5–10)	0.71 (0.43–1.13, *p*=0.160	1	0.58
Body mass index (kg/m^2^)	129	27.7 (24–31)	27.3 (24–31)	27.5 (24–31)	0.73 (0.47–1.09, *p*=0.143)	27.77	0.55
Comorbidity							
Hypertension	187	60 (47%)	36 (62%)*	96 (51%)	1.88 (1.01–3.58, *p*=0.050)	-	-
CAD	187	10 (8%)	10 (17%)	20 (11%)	2.48 (0.96–6.42, *p*=0.058)	-	-
Other cardiovascular disease	187	7 (5%)	9 (15%)*	16 (9%)	3.20 (1.13–9.42, *p*=0.029)	-	-
Neurologic	187	6 (5%)	3 (5%)	9 (5%)	1.12 (0.23–4.40, *p*=0.878)	-	-
Diabetes	187	29 (23%)	16 (28%)	45 (24%)	1.31 (0.64–2.65, *p*=0.451)	-	-
Chronic kidney failure	187	8 (6%)	6 (10%)	14 (8%)	1.75 (0.55–5.27, *p*=0.324)	-	-
Oncologic	187	9 (7.0%)	9 (16%)	18 (10%)	2.45 (0.91–6.63, *p*=0.074)	-	-
Dyslipidemia	187	18 (14.0%)	8 (14%)	26 (14%)	0.99 (0.38–2.35, *p*=0.977)	-	-
COPD	187	5 (4%)	5 (9%)	10 (5%)	2.34 (0.63–8.74, *p*=0.193)	-	-
Asthma	187	3 (2%)	2 (3%)	5 (3%)	1.50 (0.19–9.29, *p*=0.662)	-	-
Autoimmune disease	187	11 (9%)	4 (7%)	15 (8.0%)	0.79 (0.21–2.44, *p*=0.705)	-	-
Mental illness	187	6 (5%)	3 (5%)	9 (5%)	1.12 (0.23–4.40, *p*=0.878)	-	-
Smoking history							
Never	172	121 (94%)	51 (88%)	172 (92.0%)		-	-
Current or former	15	8 (6%)	7 (12%)	15 (8.0%)	2.08 (0.69–6.08, *p*=0.179)	-	-
CT findings							
Well-aerated Parenchyma	187	72 (63–81)	62 (50–75)*	68 (57–79)	0.51 (0.33–0.77, *p*=0.002)	66.1	0.65
Ground glass opacity	187	24 (15–33)	32 (24–41)*	26 (16–35)	1.95 (1.24–3.14, *p*=0.005)	29	0.64
Other: consolidation and fibrosis	187	3 (0.3–5)	6 (0.7–10)*	3 (0.2–7)	1.47 (1.08–2.04, *p*=0.017)	5.7	0.62
Laboratory findings							
White blood count, X 10^3^/μL	187	6.1 (4.2–7.9)	6.9 (4.8–9)	6.2 (4.2–8.2)	1.20 (0.87–1.67, *p*=0.259)	6.96	0.55
Lymphocytes count, X 10^3^/μL	187	1 (0.7–1.3)	0.8 (0.5–1)*	1 (0.7–1.3)	0.64 (0.40–0.96, *p*=0.047)	1	0.6
C-reactive protein, mg/dL	186	8 (2–13)	11 (5–16)*	8 (3–14)	2.15 (1.34–3.53, *p*=0.002)	8.42	0.65
Lactate dehydrogenase, U/L	149	708 (505–911)	824 (601–1047)*	754 (543–965)	1.70 (1.10–2.69, *p*=0.018)	786	0.62
Glycemia, mg/dL	187	120 (97–143)	133 (108–157)	125 (101–149)	1.22 (0.97–1.54, *p*=0.086)	138	0.59
Fibrinogen, mg/dL	70	576 (506–647)	591 (489–693)	580 (502–658)	1.25 (0.83–1.92, *p*=0.280)	597	0.53
Ferritin, ng/mL	57	1041 (588–1494)	1300 (872–1728)	1125 (671–1579)	1.53 (1.01–2.56, *p*=0.068)	1139	0.67
Procalcitonin	118	0.1 (0.05–0.1)	0.3 (0.05–0.5)	0.1 (0–0.2)	1.05 (1.00–1.12, *p*=0.120)	0.23	0.76
Troponine ng/L	109	11 (3–19)	17 (4–30)	12 (2–22)	1.00 (0.94–1.05, *p*=0.913)	22	0.65
Oxygenation support							
Standard O_2_ therapy		41 (32%)	4 (7%)*		0.27 (0.07–0.76, *p*=0.022)		
Non invasive ventilation		63 (49%)	23 (40%)		-		
Intubated		25 (19%)	31 (53%)*		3.40 (1.68–7.00, *p*=0.001)		

Descriptive statistics were reported as median and interquartile range for continuous variables and percentages (absolute numbers) for categorical variables. The logistic regression model, Odds Ratio (OR) together with the 95% confidence intervals (95% CI), and *p* values are reported for each predictor considered. Asterisks show statistical significance variables. The cut point maximizing the sum of sensitivity and specificity is shown for the continuous variable together with the corresponding area under the curve (AUC)

### Risk factors

The patients’ demographic, clinical, and laboratory characteristics along with chest CT analysis findings relative to both the whole study population and alive *vs* death subjects are listed in Table 1. When stratifying patients according to mortality, variables independently associated with mortality were age, other cardiovascular diseases except for coronary artery disease, C-reactive protein, lactate dehydrogenase levels, and IMV. Furthermore, on quantitative chest CT examination, we found a significant positive association between GGO and consolidation and fibrosis. Conversely, female sex, PaO_2_/FiO_2_, SpO_2_, lymphocytes count, standard oxygen therapy, and evidence of well-aerated lung parenchyma on chest CT scan were all inversely associated with mortality.

Table 2 enlists the patients’ demographic, clinical, and laboratory characteristics along with the chest CT analysis findings relative to both the whole population and intubated *vs* non-intubated patients. Among our cohort of 187 patients, 29 patients were excluded from this analysis because classified as “do-not-intubate” subjects, i.e., patients deemed ineligible for intubation in case of CPAP or BiPAP failure. Variables independently associated with intubation were C-reactive protein, lactate dehydrogenase levels, and white blood cell count. On quantitative chest CT analysis, GGO, consolidation, and fibrosis resulted positively associated with intubation, while PaO_2_/FiO_2_, SpO_2_, lymphocyte count, and well-aerated parenchyma were inversely associated with intubation. Lastly, for each logistic regression and for the continuous variables the cut points maximizing the best predictive value along with their corresponding area under the curve (AUC) are shown in Tables 1 and 2.

**Table 2:** Patient characteristics and clinical, laboratory, and CT findings relative to intubated and non-intubated patients

Variable	All patients (*n*= 158)	Non-intubated *n*=102	Intubated *n*=56	Total (158)	OR (univariable)	Cut point	AUC
Age (year)	158	63 (55–71)	62 (56–68)	62 (55–70)	0.90 (0.59–1.37, *p*=0.619	64	0.53
Sex							
Male		72 (71)	46 (82)	118 (75)		-	-
Female		30 (29)	10 (18)	40 (25)	0.52 (0.22–1.14, *p*=0.113)	-	-
PaO_2_/FIO_2_	154	280 (246–314)	223 (168–278)*	264 (21–312)	0.43 (0.26–0.68, *p*< 0.001)	245	0.68
SpO_2_ (%)	156	93 (90–95)	88 (79–97)*	92 (88–96)	0.55 (0.39–0.75, *p*< 0.001)	88.4	0.66
Symptom onset	151	7 (5–10)	7 (5–10)	7 (5–10)	0.92 (0.56–1.49, *p*=0.733)	1	0.51
Body mass index (kg/m^2^)	107	27.3 (24–31)	27.7 (25–31)	27.5 (24–31)	0.96 (0.64–1.45, *p*=0.859)	27.6	0.53
Comorbidity							
Hypertension	158	53 (52.0%)	25 (45%)	78 (49%)	0.75 (0.39–1.43, *p*=0.379)	-	-
Coronary artery disease	158	11 (11%)	6 (11%)	17 (11%)	1.00 (0.34–2.66, *p*=0.996)	-	-
Other cardiovascular disease	158	6 (6%)	3 (5%)	9 (6%)	0.91 (0.19–3.58, *p*=0.892)	-	-
Neurologic disease	158	3 (3%)	5 (9%)	8 (5%)	3.24 (0.76–16.28, *p*=0.118)	-	-
Diabetes	158	23 (23%)	11 (20%)	34 (22%)	0.84 (0.36–1.85, *p*=0.671)	-	-
Chronic kidney failure	158	6 (6%)	2 (4%)	8 (5%)	0.59 (0.08–2.67, *p*=0.530)	-	-
Oncologic	158	8 (8%)	6 (11%)	14 (9%)	1.41 (0.44–4.28, *p*=0.545)	-	-
Dyslipidemia	158	16 (16%)	7 (13%)	23 (15%)	0.77 (0.28–1.93, *p*=0.588)	-	-
COPD	158	5 (5%)	0 (0.0)	5 (3%)	Not estimable	-	-
Asthma	158	4 (4%)	0 (0.0)	4 (4%)	Not estimable	-	-
Autoimmune disease	158	7 (7%)	5 (9%)	12 (8%)	1.33 (0.38–4.38, *p*=0.640)	-	-
Mental illness	158	4 (4%)	2 (4%)	6 (4%)	0.91 (0.12–4.81, *p*=0.912)	-	-
Smoking history	158						
Never		95 (93%)	51 (91%)	146 (92%)	-	-	-
Current or former		7 (7%)	5 (9%)	12 (8%)	1.33 (0.38–4.38, *p*=0.640)	-	-
CT findings							
Well-aerated parenchyma	158	74 (64–84)	63 (52–74)*	68 (58–79)	0.37 (0.22–0.58, *p*< 0.001)	67.7	0.7
Ground glass opacity	158	23 (14–32)	33 (24–42)*	25 (16–35)	2.50 (1.52–4.26, *p*< 0.001)	28.4	0.67
Other: consolidation and fibrosis	158	3 (0.7–5)	7 (2.2–11)*	3 (0.3–7)	2.13 (1.46–3.26, *p*< 0.001)	5.8	0.68
Laboratory findings							
White blood count, X 10^3^/μL	158	5.9 (4.3–7.4)	6.6 (4–9)*	6.1 (4.1–8.1)	1.50 (1.06–2.18, *p*=0.026)	7.03	0.58
Lymphocyte count, X 10^3^/μL	158	1.1 (0.8–1.4)	0.9 (0.7–1.2)*	1 (0.7–1.3)	0.56 (0.33–0.90, *p*=0.024)	1.06	0.61
C-reactive protein, mg/dL	158	7 (3–12)	12 (5–19)*	8 (2–14)	3.37 (1.92–6.22, *p*< 0.001)	11.3	0.68
Lactate dehydrogenase, U/L	158	694 (494–894)	870 (641–1091)*	766 (560–972)	2.20 (1.39–3.67, *p*=0.001)	796	0.69
Glycemia, mg/dL	158	113 (91–134)	133 (113–152)	121 (99–142)	1.11 (0.88–1.42, *p*=0.373)	128	0.61
Fibrinogen, mg/dL	63	583 (520–646)	577 (490–664)	581 (506–656)	0.91 (0.59–1.39, *p*=0.667)	538	0.58
Ferritin, ng/mL	48	1082 (641–1523)	1330 (563–2096)	1111.5 (629–1594)	1.63 (0.92–3.03, *p*=0.098)	1514	0.61
Procalcitonin	103	0.1 (0.05––0.2)	0.2 (0.05–0.4)	0.1 (0–0.2)	1.04 (1.00–1.13, *p*=0.154)	0.14	0.73
Troponin ng/L	93	11 (4–18)	12 (1–23)	11 (3–20)	0.99 (0.92–1.03, *p*=0.751)	17	0.58

Descriptive statistics were reported as median and interquartile range for continuous variables and percentages (absolute numbers) for categorical variables. The logistic regression model, odds ratio (OR) together with the 95% confidence intervals (95% CI), and *p* values are shown for each predictor considered. Asterisks show statistical significance variables. The cut point maximizing the sum of sensitivity and specificity is shown for the continuous variable together with the corresponding area under the curve (AUC)

### RF algorithm

**Fig. 2:**
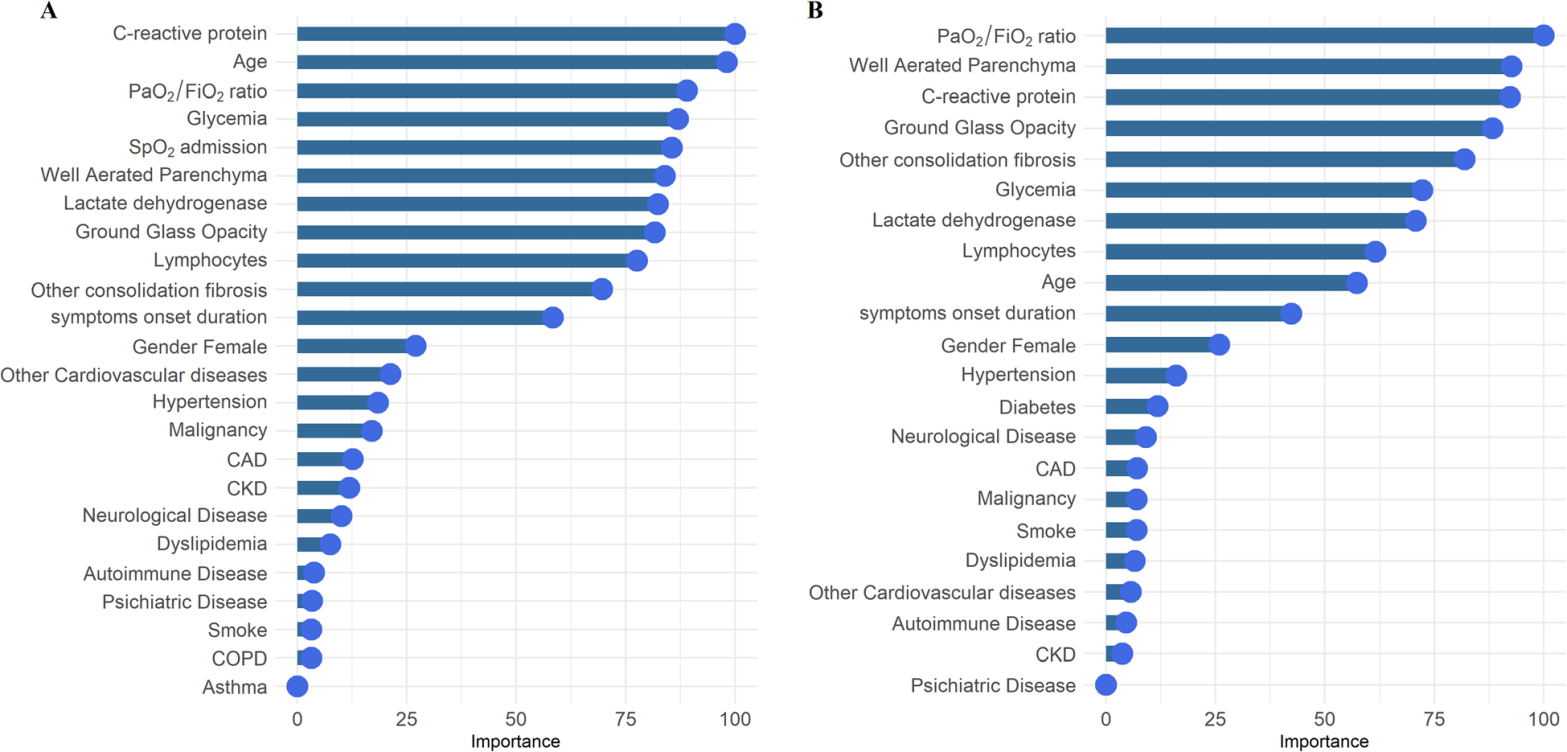
Important predictor variables (importance > 50) in the random forest (RF) models for intra-hospital mortality (**a**) and intubation prediction (**b**)

**Fig. 3:**
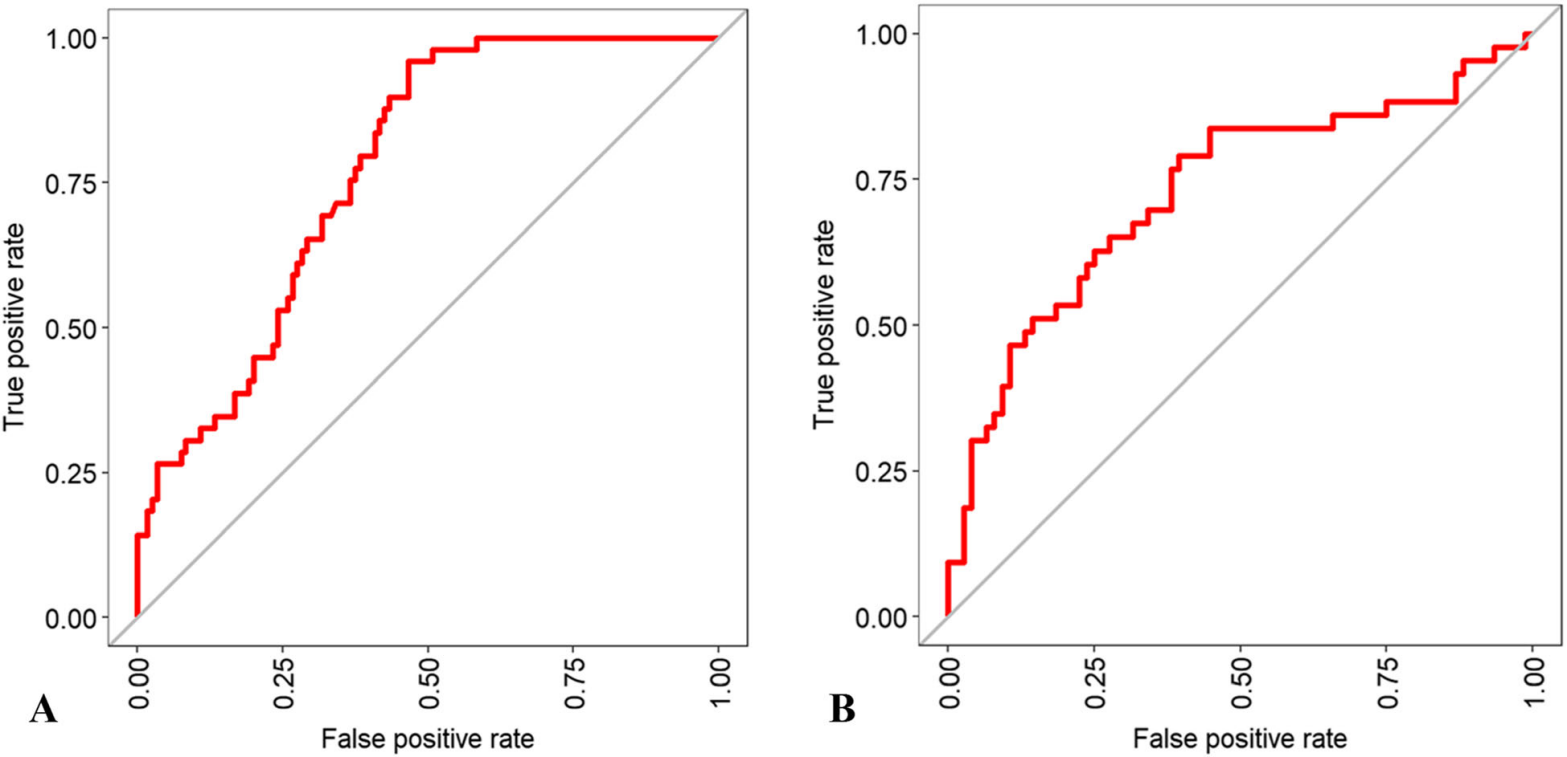
AUC of the RF models. **a**) The RF model balanced accuracy in predicting intra-hospital mortality is 0.89 (*κ* = 0.72; AUC = 0.73). **b** The RF model balanced accuracy in predicting intubation is 0.9 (*κ* = 0.75; AUC = 0.74)

**Fig. 4:**
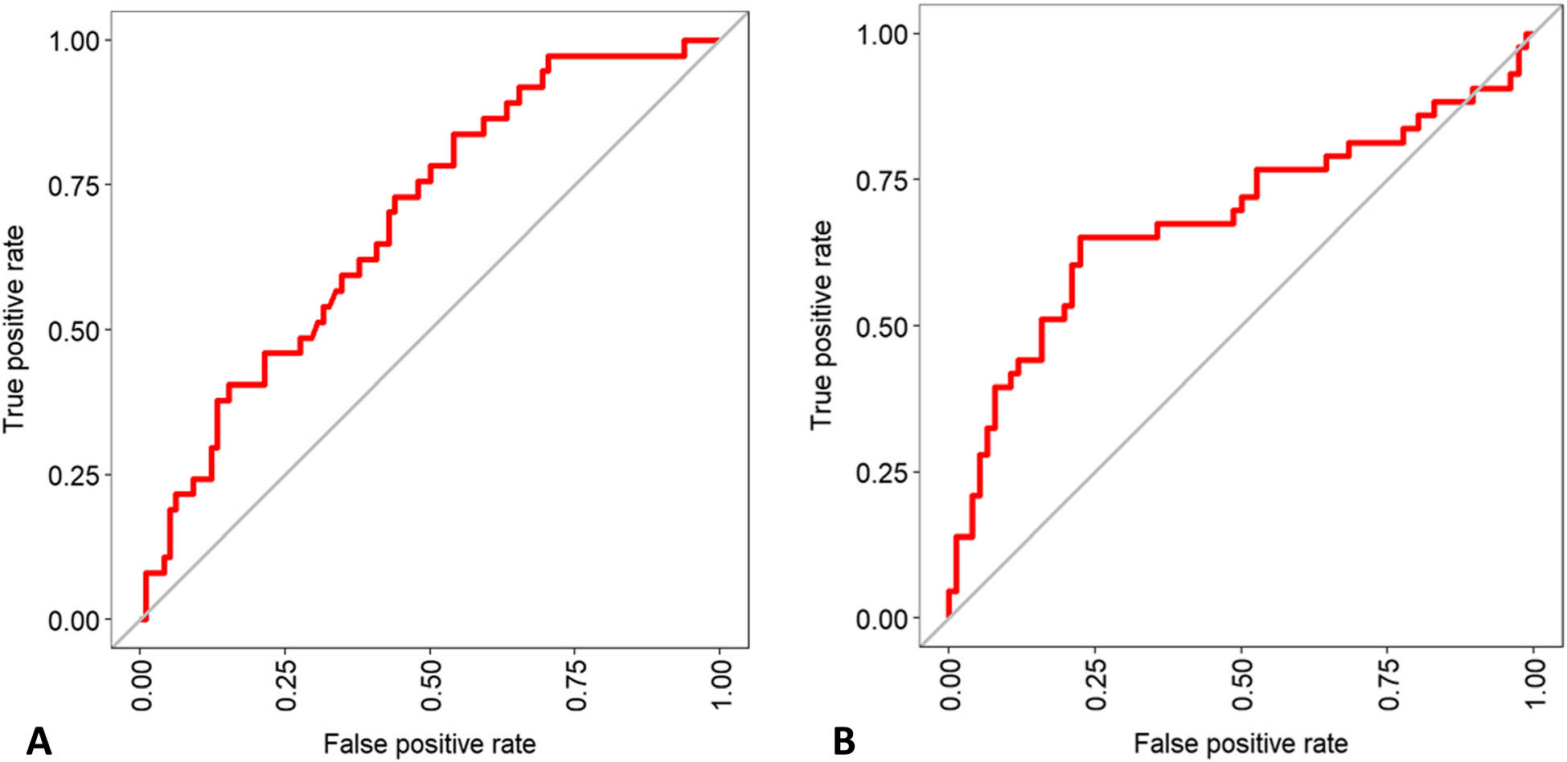
RF model performance without CT parameters. **a** The RF model balanced accuracy in predicting intra-hospital mortality is 0.75 (*κ* = 0.1). **b** The RF model balanced accuracy in predicting intubation is 0.69 (*κ* = 0.29)

We next evaluated the importance of the variables encompassed in the RF algorithms for intra-hospital mortality and intubation prediction. In the RF model for mortality prediction, the most important nodes (importance > 50) were C-reactive protein, age, PaO_2_/FiO_2_, glycemia, SpO_2_ on admission, a well-aerated parenchyma, lactate dehydrogenase, GGO, lymphocytes, other consolidation/fibrosis, and symptom onset (Fig. 2A). In the RF tree model for intubation prediction, the most important nodes (importance > 50) were PaO_2_/FiO_2_, a well-aerated parenchyma, C-reactive protein, GGO, other consolidation/fibrosis, glycemia, lactate dehydrogenase, lymphocytes, and age (Fig. 2B). The variables having more than 10% of missing values were excluded from the predictive tool (i.e., BMI, fibrinogen, procalcitonin, ferritin, and troponin). The balanced accuracy in predicting intra-hospital mortality was 0.89 (*κ* value = 0.72; AUC = 0.73) (Fig. 3A), whereas the balanced accuracy in predicting intubation was 0.9 (*κ* value = 0.75; AUC = 0.74) (Fig. 3B). When the quantitative CT analysis variables were removed from both RF models, the accuracy of the model predicting intra-hospital mortality dropped to 0.75, whereas that of the model predicting intubation fell to 0.69 (Fig. 4A, B).

## Discussion

The main findings of our investigation can be summarized as follows: (*i*) in our cohort of COVID-19 patients, elderly male subjects with comorbidities, such as other cardiovascular diseases, intubated for severe hypoxemic ARF associated with a major inflammatory response and a widespread pulmonary involvement on chest CT were at increased risk of intra-hospital mortality; (*ii*) excluding those patients classified as “do-not-intubate,” subjects with severe hypoxemic ARF experiencing increased inflammatory response and poor lung aeration on chest CT were at increased risk of intubation; and (*iii*) our novel RF algorithms performed well in predicting intra-hospital mortality and intubation in our study population.

The intra-hospital mortality rate of critically ill patients admitted for COVID-19 has been reported to range from 17 to 67% [18]. Well recognized risk factors associated with low survival or poor outcome in ICU are: male gender, increasing age, comorbidities such as diabetes, hypercholesterolemia, chronic obstructive pulmonary disease, IMV at high positive end-expiratory pressure, low PaO_2_/FiO_2_ on ICU admission, high SOFA score, acute kidney injury, reduced respiratory system compliance, late pulmonary infections, and cardiovascular complications [14, 19].

In our setting, intra-hospital mortality rate was 31%, in line with previous reports [18]. Risk factors for intra-hospital mortality identified in our cohort confirmed all previously reported predictors [10–13]. Furthermore, increased C-reactive protein and lactate dehydrogenase and reduced lymphocyte count were all associated with increased mortality in our study population, which is in good agreement with previous data showing a positive association between severity/mortality rate of COVID-19 illness and biomarkers such as C-reactive protein, lactate dehydrogenase, and lymphopenia [12, 13]. Of note, the intubation rate in our study was 35.4%, which is consistent with the IMV incidence range among COVID-19 patients (12–33.1%) [20–22].

Older age, BMI, comorbidities—i.e., hypertension, diabetes, and cardiovascular diseases—, shortness of breath, SpO_2_ < 90%, and increased respiratory rate are well-known predictors of intubation in patients admitted for COVID-19 [9]. In our cohort of patients, we confirm that reduced SpO_2_ and/or PaO_2_/FiO_2_ are risk factors for IMV. We also show that increased C-reactive protein and lactate dehydrogenase serum concentrations and elevated total white blood cell count are predictors of intubation, which is in good agreement with previous reports demonstrating the association between the aforementioned biomarkers and illness severity [12, 13].

Lung aeration loss on chest CT scan has been previously shown to be an independent predictor of death and ICU admission in COVID-19 patients suffering from hypoxemic ARF [17, 23]. Fittingly, we found that reduced aerated lung volume and increased GGO and/or consolidation and fibrosis are indicators of poor outcome and intubation. In this regard, Colombi et al. [23] have previously demonstrated an association between mortality and exudative consolidation, which may be suggestive of concomitant bacterial infection associated with death in COVID-19 patients [24].

To date, several models have been proposed to estimate the risk of COVID-19 patients to be hospitalized or to experience a poor outcome from the infection in order to assist medical staff in triaging patients when allocating limited healthcare resources [25]. With particular regard to predictive models for mortality and progression to a more severe or critical condition, the most frequently used predictors include comorbidities, age, sex, lymphocyte count, C-reactive protein, body temperature, creatinine, and imaging features [25]. The discrimination of these models ranged from 0.68 to 0.98 for intra-hospital mortality [26] and from 0.73 to 0.99 for worsening to a more critical state [27].

Here, we propose two novel RF models developed by including all the demographic, clinical, hemato-chemical, and radiological variables from our cohort of COVID-19 patients having less than 10% of missing data. The predictive balanced accuracy was high for both RF models, probably because the number of nodes exceeding an importance of 50 was very high for each algorithm. Among the items included in our RF algorithms for prediction of mortality and intra-hospital intubation, blood glucose and the symptom onset duration were the two factors that, in addition to the predictors listed above, showed an importance > 50.

Our findings are in keeping with recent results suggesting that hyperglycemia, even in the absence of frank diabetes, is associated with a negative outcome compared to normoglycemic individuals as well as to those with pre-existing diabetes and COVID-19 [28]. Also, the symptom onset duration was confirmed to be a poor outcome predictor, being a fever lasting more than 7 days from onset of illness associated with increased ICU admission [29].

Although our study confirms with an innovative approach (i.e., RF) the risk factors of intubation and mortality found in the recent literature, it has several limitations. First, due the retrospective nature of the present single-center study, our results lack of generalizability. Second, no power sample was estimated for RF model accuracy assessment. Thus, no definitive conclusions can be drawn on the precision of our algorithm for intubation and intra-hospital mortality prediction. Third, some variables were excluded during the algorithm construction due to missing data occurrence > 10% and the fact that other laboratory values, such as PaCO_2_, creatinine, and D-Dimer were not collected. Lastly, our prediction models were not validated before the present investigation. Therefore, a future prospective investigation addressing the validation of our models is clearly needed.

## Conclusions

In our cohort of COVID-19 patients, we confirmed all demographic, clinical, hemato-chemical, and radiologic predictors of adverse outcomes previously reported. In addition, our innovative RF models based on the risk factors identified in our subset showed a good level of accuracy in predicting intra-hospital mortality and intubation. Thus, this approach may help to accurately identify patients at higher risk of deterioration, which would be particularly important in case of disproportion between resources and demand. Our results await further confirmation in larger multicentric prospective studies.

## Supplementary Information

**Additional file 1.** Supplementary information

## Abbreviations

ARF: Acute respiratory failure
BMI: Body mass index
BiPAP: Bilevel positive airway pressure
CAD: Coronary artery disease
COPD: Chronic obstructive pulmonary disease
CPAP: Continuous positive airway pressure
COVID-19: Coronavirus disease 19
CT: Computerized tomography
DICOM: Digital Imaging and Communications in Medicine
DT: Decision trees
GGO: Ground glass opacity
HU: Hounsfield
ICU: Intensive care unit
IMV: Invasive mechanical ventilation
PACS: Picture Archiving and Communication System
PaO_2_/FiO_2_: Arterial oxygen pressure on inspired oxygen fraction ratio
RF: Random forest
RT-PCR: Real-time reverse transcriptase-polymerase chain reaction
SARS-CoV-2: Severe acute respiratory syndrome coronavirus 2
SOFA: Sequential organ failure assessment
SpO_2_: Peripheral oxygen saturation
