# Supplementary material for: Predictors of intubation and mortality in COVID-19 patients: a retrospective study
Source: J Anesth Analg Crit Care. 2021 Nov 27;1:19. doi: 10.1186/s44158-021-00016-5 (PMC8626752; doi:10.1186/s44158-021-00016-5)
Supplement: Supplementary file 1 — Additional file 1. Supplementary information [file 44158_2021_16_MOESM1_ESM.docx]

**Title: Predictors of intubation and mortality in COVID-19 patients: a retrospective study**

Tiziana Cena, MD^1^, Gianmaria Cammarota, MD^1*^, PhD^1^, Danila Azzolina, MD^2,^ Michela Barini, MD^3^, Simona Bazzano^1^, Domenico Zagaria, MD^3^, Davide Negroni, MD^3^, Luigi Castello, MD^4^, Alessandro Carriero, MD^2,3^, Francesco Della Corte, MD^1,2^, Rosanna Vaschetto, MD, PhD^1,2^.

^1^Azienda Ospedaliero Universitaria “Maggiore della Carità”, Anestesia e Terapia Intensiva, Novara, Italy.

^2^Università del Piemonte Orientale, Dipartimento di Medicina Traslazionale, Novara, Italy.

^3^Azienda Ospedaliero Universitaria “Maggiore della Carità”, Radiologia, Novara, Italy.

^4^Azienda Ospedaliero Universitaria “Maggiore della Carità”, Medicina d’Urgenza, Novara, Italy

**Supplementary Information**

**Quantitative CT analysis**

Chest CT was performed using 128 slice multidetector row TC scanners. TC acquisition and reconstruction parameters were as follows: tube voltage, 120 kV; tube current modulation, 226 mAs; spiral pitch factor, 1.08; collimation width 0.625, matrix 512 (mediastinal window) and 768 (lung window). All images were reconstructed with 1 mm slice thickness range using both sharp kernels (B70f) with a standard lung window (1500 width; -500 centers) and medium-soft kernels (B40f) with a soft-tissue window (300 widths; 40 centers). The images in Digital Imaging and Communications in Medicine (DICOM) extension files were transferred to the Picture Archiving and Communication System (PACS) of our institution and then analyzed into a workstation equipped with two 35 × 43 cm monitors (produced by Eizo, with 2048 × 1536 matrix).

The open-source 3D Slicer Software for the lung parenchyma segmentation was the version 4.10.2, https://www.slicer.org)^1^ using the software tools called “Segment Editor” and “Segment Quantification”. The hardware used was an HP Pavilion, processor: AMD Ryzen 5-3500U (2.1 GHz - 4 MB L3), SSD: 256 GB, RAM: 8 GB, graphic card: AMD Radeon Vega 8. Due to the patient clinical condition, the maximum expiratory scan could not be obtained.

**Definition of CT findings and analysis**

Specific density thresholds expressed according to the Hounsfield (HU) scale values were set for each of the three main characteristics analyzed on lungs affected by COVID-19 pneumonia: 1) well-aerated parenchyma; 2) interstitial lung disease, including both ground glass opacities (GGO) and crazy paving; and 3) parenchymal consolidation.

GGO was defined as hazy increased opacity of the lung, with preservation of bronchial and vascular margins [1]. Crazy-paving pattern was defined as GGO associated with interlobular septal thickening [2]. Consolidation was defined as a homogeneous increase in pulmonary parenchymal attenuation obscuring the margins of the vessels and airway walls, represented by replacement of alveolar air by fluid, blood, pus, cells, or other substances. From the analysis of previous studies, the HU values related to the well-aerated lung parenchyma were defined within a threshold between -950 and -700 HU [3]. As recommended by the literature [4], the threshold from -700 to -250 HU was selected to isolate the interstitial involvement of the disease—including typical COVID-19 pneumonia features as GGO and crazy paving—from the surrounding lung parenchyma. Optimal isolation was achieved thanks to the substantial difference in densitometric values between the air and the lung. With regard to the lung consolidation densitometry, the thresholding selection was set by combining visual assessment and the proposed [5] HU range i.e., -250 HU up to + 250 HU.

**WEB application**

The random forest (RF) predictive tool was developed in a web application written in R programming language and RStudio. The app development was performed within a Shiny framework. Shiny apps have two components: a user-interface (ui) script and a server script. The ui script controls the layout and appearance of an application. In ui.R, functions are used for laying out the user interface. The server.R script contains the instructions that the computer needs to build an application. In server.R, the functions are used for the application development.
The RF application can be initiated locally from any computer that has either an R installed or uninstalled. The App is tested on several browsers, such as Chrome, Internet Explorer, Firefox, and Opera, both in Windows and Linux operating systems. The application is available on a cloud hosted by the shinyapps.io server <https://usruniupo.shinyapps.io/covid_19_predictioner/>.

**Statistical Analysis**

The sample size was determined for an area under the curve (AUC) estimation problem to validate the predictive ability of a predictive model. The procedure is based on optimizing the determined sample size by defining a specific margin of error d and a 1-𝛼 confidence level. The formula applied is as follows, as suggested in the literature [6]:

n=(Z_(a/2)^2 V(AUC))/d^2

In the equation above (AUC) can be estimated as:

V(AUC)=(0.0099×e^(-a^2/2) )×(6a^2+16)

Where a=φ^(-1) (AUC)×1.414" e " φ^(-1) is the inverse of cumulative rd.

Several scenarios for calculating the sample size were defined by setting: *i*) a confidence level of 1-𝛼 al 95%, *ii*) an error margin from 0.05 to 0.08, *iii*) an AUC value between 0.75 and 0.85.

The results show that a sample of 187 patients ensures a predictive ability near to 0.8 with a margin of error in the sample estimates d=0.05. The calculation has been performed via R software version 3.4.2.

Reference List

1. Hansell DM, Bankier AA, MacMahon H, McLoud TC, Muller NL, Remy J (2008) Fleischner Society: glossary of terms for thoracic imaging. Radiology 3(246):697-722. https://doi.org/10.1148/radiol.2462070712

2. Caruso D, Polidori T, Guido G, Nicolai M, Bracci B, Cremona A, Zerunian M, Polici M, Pucciarelli F, Rucci C, Dominicis C, Girolamo MD, Argento G, Sergi D, Laghi A (2020) Typical and atypical COVID-19 computed tomography findings. World J Clin Cases 15(8):3177-3187. https://doi.org/10.12998/wjcc.v8.i15.3177

3. Sverzellati N, Milanese G, Milone F, Balbi M, Ledda RE, Silva M (2020) Integrated Radiologic Algorithm for COVID-19 Pandemic. J Thorac Imaging 4(35):228-233. https://doi.org/10.1097/RTI.0000000000000516

4. Tabatabaei SMH, Talari H, Gholamrezanezhad A, Farhood B, Rahimi H, Razzaghi R, Mehri N, Rajebi H (2020) A low-dose chest CT protocol for the diagnosis of COVID-19 pneumonia: a prospective study. Emerg Radiol 6(27):607-615. https://doi.org/10.1007/s10140-020-01838-6

5. Rorat M, Jurek T, Simon K, Guzinski M (2021) Value of quantitative analysis in lung computed tomography in patients severely ill with COVID-19. PLoS One 5(16):e0251946. doi: <https://doi.org/10.1371/journal.pone.0251946.g007>

6. Hajian-Tilaki K (2014) Sample size estimation in diagnostic test studies of biomedical informatics. J Biomed Inform 48):193-204. <https://doi.org/10.1016/j.jbi.2014.02.013>
